# AglH, a thermophilic UDP-*N*-acetylglucosamine-1-phosphate:dolichyl phosphate GlcNAc-1-phosphotransferase initiating protein *N*-glycosylation pathway in *Sulfolobus acidocaldarius*, is capable of complementing the eukaryal Alg7

**DOI:** 10.1007/s00792-016-0890-2

**Published:** 2016-11-07

**Authors:** Benjamin H. Meyer, Hosam Shams-Eldin, Sonja-Verena Albers

**Affiliations:** 1 Molecular Biology of Archaea, Institute of Biology, University of Freiburg, Schaenzlestrasse 1, 79211 Freiburg, Germany; 2 Institute of Virology, Hans-Meerwein-Str. 2, 35043 Marburg, Germany; 3 Division of Molecular Microbiology, School of Life Sciences, University of Dundee, Dundee, DD1 5EH UK

**Keywords:** *N*-Glycosylation, Crenarchaea, *Sulfolobus*, Glycosylation, Dolichol phosphate, Alg7, AglH

## Abstract

AglH, a predicted UDP-GlcNAc-1-phosphate:dolichyl phosphate GlcNAc-1-phosphotransferase, is initiating the protein *N*-glycosylation pathway in the thermoacidophilic crenarchaeon *Sulfolobus acidocaldarius*. AglH successfully replaced the endogenous GlcNAc-1-phosphotransferase activity of Alg7 in a conditional lethal *Saccharomyces cerevisiae* strain, in which the first step of the eukaryal protein *N*-glycosylation process was repressed. This study is one of the few examples of cross-domain complementation demonstrating a conserved polyprenyl phosphate transferase reaction within the eukaryal and archaeal domain like it was demonstrated for *Methanococcus voltae* (Shams-Eldin et al. 2008). The topology prediction and the alignment of the AglH membrane protein with GlcNAc-1-phosphotransferases from the three domains of life show significant conservation of amino acids within the different proposed cytoplasmic loops. Alanine mutations of selected conserved amino acids in the putative cytoplasmic loops II (D_100_), IV (F_220_) and V (F_264_) demonstrated the importance of these amino acids for cross-domain AlgH activity in in vitro complementation assays in *S. cerevisiae*. Furthermore, antibiotic treatment interfering directly with the activity of dolichyl phosphate GlcNAc-1-phosphotransferases confirmed the essentiality of *N*-glycosylation for cell survival.

## Introduction

All living cells exhibit an outer surface covered with an array of glycans. These glycans are either loosely attached or covalently linked to surface proteins or lipids. Protein glycosylation is one of the most common posttranslational protein modifications found in all three domains of life (Larkin and Imperiali 2011). In particular, protein *N*-glycosylation is widely distributed in Eukarya and Archaea whereas it is rarely found in Bacteria. In these systems, the biosynthesis of *N*-linked oligosaccharides is initiated by the transfer of a sugar(-1-phosphate) residue from a nucleotide-activated sugar onto the lipid carrier dolichyl phosphate (DolP) or undecaprenyl phosphate (UndP), respectively. The fully assembled lipid-linked glycan is then transferred to a specific Asn (*N*) residue within a target protein.

Eukaryotic protein *N*-glycosylation is initiated by the GlcNAc-1-phosphate transferase Alg7/Dpagt1 (Lehrman 1991; Mclachlan and Krag 1992), which converts UDP-GlcNAc and DolP into UMP and DolPP-GlcNAc. This step is essential in Eukarya and defined mutations in the human Dpagt1 cause severe clinical phenotypes, leading to lethal diseases (Jaeken and Matthijs 2007; Wurde et al. 2012). DolPP-GlcNAc acts as a primer for the elongation of the *N*-glycans by specific glycosyltransferases (GTases), sequentially transferring individual sugars from corresponding nucleotide- or lipid phosphate-activated precursors (Burda and Aebi 1999).

The bacterial protein *N*-glycosylation pathway from *Campylobacter jejuni* starts with the transfer of diacetamido bacillosamine-1-P from UDP-2,4-diacetamido bacillosamine onto the lipid carrier UndP by the phosphotransferase PglC (Glover et al. 2006). Similar to the initiation of protein *N*-glycosylation, the biosynthesis of several other bacterial glycoconjugates starts at the cytoplasmic site of the cell membrane with the formation of an UndPP-linked monosaccharide. Examples include the biosynthesis of O-antigen polymers (Meier-Dieter et al. 1992; Samuel and Reeves 2003; Schmidt et al. 1976), the capsular antigens (Masson and Holbein 1985; Troy et al. 1975; Whitfield 2006), lipopolysaccharide (Alexander and Valvano 1994; Schmidt et al. 1976), teichoic acids (Brown et al. 2008; Ginsberg et al. 2006; Mancuso and Chiu 1982) and the peptidoglycan (Bouhss et al. 2008; Bugg and Brandish 1994; Typas et al. 2012). The initiation step is meditated by different classes of membrane-bound UDP-hex(NAc)-1-phosphate:polyprenyl phosphate sugar-1-phosphotransferase enzymes, which use UndP as an acceptor but differ in their specificity for the nucleotide sugar donor. The bacterial enzyme WecA (formerly known as Rfe) catalyses the first step of biosynthesis of many LPS O-antigens and capsular K-antigens by the transfer of GlcNAc-1-P residue from UDP-GlcNAc onto UndP (Amor and Whitfield 1997).

In Archaea, *N*-glycosylation pathways for three different euryachaea and one crenarchaeon have been described [for review see (Jarrell et al. 2014)]. In contrast to Eukarya and Bacteria, most of the euryarchaeal *N*-glycans characterised to date, i.e. *Hfx. volcanii*, *Haloarcula marismortui*, *Methanococcus voltae*, and *Pyrococcus furiosus*, are assembled on DolP lipid carrier (Calo et al. 2011; Chang et al. 2015; Guan et al. 2010; Larkin et al. 2013), with two exceptions. *Methanothermus fervidus* assemble their *N*-glycans on DolPP (Hartmann and König 1989) and *Hbt. salinarum*, which synthesizes two distinct *N*-glycans, assembles one on DolP and the second on DolPP (Paul and Wieland 1987). A recent comparison of archaeal lipid-linked oligosaccharides revealed a difference between eury- and crenarchaeota. Indeed, selected species of the euryarchaeota possessed DolP-linked glycans, whereas the crenarchaeota *Pyrobaculum calidifontis* and *Sulfolobus solfataricus* assemble their *N*-glycans on DolPP (Taguchi et al. 2016). Different steps of the *N*-glycosylation pathway of the closely related crenarchaeon *Sulfolobus acidocaldarius* have been studied in detail (Meyer and Albers 2013). Here, a tribranched hexasaccharide, composed of two GlcNAc, two terminal Man, one sulfoquinovose and one terminal Glc are found as the *N*-glycan linked to the S-layer protein (Peyfoon et al. 2010). Interestingly, the basal structure of this archaeal *N*-glycan (GlcNAc_2_Man) resembles this of the eukaryal one and the biosynthesis might rely on homologs to the eukaryal ones.

Unlike in euryarchaeota, the initiation step of the protein *N*-glycosylation process in thermophilic crenarchaeota has not yet been elucidated. In the present study, we identified a candidate enzyme for the initiation step of the protein *N*-glycosylation pathway in *S. acidocaldarius* by homology searches. The identified AglH (Saci0093) is able to restore *N*-glycosylation in a conditional lethal *Saccharomyces cerevisiae alg*7 mutant. Various attempts to delete *aglH* in *S. acidocaldarius* were unsuccessful, and the use of tunicamycin, a specific inhibitor of UDP-HexNAc-1-phosphate:polyprenol phosphate HexNAc-1-phosphotransferases, as well as the treatment with bacitracin interfering with the regeneration of DolP, revealed the essentiality of AglH in *S. acidocaldarius*.

## Materials and methods

### Strains and growth conditions

*Sulfolobus acidocaldarius* background strain MW001 (Δ*pyrE*) (Wagner et al. 2012) and the genetically-modified strains *S. acidocaldarius* BM-A120-124 (see Table 1) were grown under shaking conditions in Brock medium at 79 °C, pH 3, and supplemented with 0.1% w/v NZ amine and 0.1% w/v dextrin as carbon and energy source (Brock et al. 1972). For the uracil auxotrophic strains, the growth medium was supplemented with 10 mg ml^−1^ uracil. Gelrite (0.6%) plates were supplemented with the same nutrients, with the addition of 10 mM MgCl_2_ and 3 mM CaCl_2_. For second selection plates, 10 mg ml^−1^ uracil and 100 mg ml^−1^ 5-fluoroorotic acid (5-FOA) were added. Growth of the cultures was monitored by measuring the optical density at 600 nm. The *S. cerevisiae* strains YPH499-HIS-GAL-ALG7 (Mazhari-Tabrizi et al. 1999) used as a control and recipient strain, as well as the complementation strains were grown either in SD medium (2% dextrose, 0.17% Bacto yeast nitrogen base) (repression medium) or in SGR-His medium (4% galactose, 2% raffinose, 0.17% Bacto yeast nitrogen base, 0.5% ammonium sulphate) (non-selective medium). The 1.5% agar plates containing either SGR or SD medium were used for the complementation assay.

**Table 1:** Strains used in this study

Strain	Genotype	References
MW001	*S. acidocaldarius* DSM 639 Δ*pyrE*	Wagner et al. (2012)
BM-A120-124	MW001 strain with the integrated plasmid pSVA1229	This study
YPH499-HIS-GAL-ALG7	Mat *a*; ura3-52; lys2-801^amber^; ade2 ± 101^ochre^ trpl-D63; his3-D200; leu2-D1, GAL-ALG7	Mazhari-Tabrizi et al. (1999)

### Growth studies with bacitracin and tunicamycin

Pre-cultures grown to an OD_600_ of 0.4–0.5 were used to inoculate 5 ml of fresh Brock medium (0.1% w/v NZ amine and 0.1% w/v dextrin) to a start OD of 0.01. Directly with inoculation or after reaching exponential growth phase, different concentrations of the antibiotics bacitracin (0.6, 1.2, and 2.4 mM) and tunicamycin (2, 6, 16, and 32 µg/ml) were added. Growth was monitored by measuring the OD_600_ over a time period of 80 h.

### Construction of a Δ*aglH* deletion

A markerless deletion mutant would represent an important tool to analyse whether the predicted UDP-GlcNAc-1-phosphate:polyprenyl phosphate GlcNAc-1-phosphotransferase (AglH) indeed catalyses the first step of the protein *N*-glycosylation process. A gene deletion plasmid was used for homologous recombination as described previously (Wagner et al. 2009). To construct the deletion plasmid, 800–1000 bp of sequence up- and downstream of *saci0093* was PCR amplified. *Apa*I and *Bam*HI restriction sites were introduced at the 5ʹ ends of the upstream forward primer (1842) and the downstream reverse primer (1845), respectively. The upstream reverse primer (1843) and the downstream forward primer (1844) were each designed to incorporate 15 bp of the reverse complement strand of the other primer, resulting in a 30 bp overlapping stretch. The up- and downstream fragments were fused by overlapping PCR, using the 3ʹ ends of the up- and downstream fragments as primers. The resulting PCR product was cloned into plasmid pSAV407 using the restriction enzymes *Apa*I and *Bam*HI, yielding the plasmid pSVA1229. The plasmid was transformed into *Escherichia coli* DH5α cells, which were subsequently plated on LB agar containing 50 mg ml^−1^ ampicillin. The plasmid was confirmed by sequencing. To avoid restriction in *S. acidocaldarius*, the plasmid was methylated by transformation in *E. coli* ER1821 cells containing pM.EsaBC4I, which expresses a methylase (obtained from NEB).

### Cloning of *aglH*_FLAG_ into a *S. cerevisiae expression* vector

To verify the proposed enzymatic function of AglH we reasoned that *aglH* might be able to complement a conditional lethal yeast *alg*7 mutant, as has previously been demonstrated for the human Alg7 (Eckert et al. 1998) and the AglH of *M. voltae* (Shams-Eldin et al. 2008). We, therefore, cloned *aglH* fused to a FLAG-tag sequence into the pRS426-MET (Mumberg et al. 1995) expression vector. The *aglH* gene was PCR amplified with forward primer (1755) and reverse primer (1756), introducing *Eco*RI and *Xho*I restriction sites, respectively. The 1756 primer incorporated the FLAG-tag sequence upstream of the *Xho*I restriction site. The digested PCR fragment was cloned into the expression vector pRS426-MET (Mumberg et al. 1995) linearised with the restriction enzymes *Eco*RI and *Xho*I, creating plasmid pSAV1212 (Table 3). The plasmid was transformed into *E. coli* DH5α, and was confirmed by sequencing.

### Oligonucleotide-directed mutagenesis of *aglH*_FLAG_

Plasmid pSVA1212 was used as a template for the oligonucleotide-directed mutagenesis of *aglH*_FLAG_. In general, a PCR with a specific forward and reverse primers (Table 2) carrying the nucleotide exchange were used to amplify the gene. The resulting PCR products were digested with *Dpn*I to exclude the methylated template plasmid pSVA1229. After purification, each construct was transformed into *E. coli* DH5α, and platted on selective LB plates containing 50 mg ml^−1^ ampicillin. The exchange of the nucleotides was confirmed by sequencing of the entire *aglH*_FLAG_ gene.

**Table 2:** Oligonucleotide primers used in this study

Primer number	Primer sequence (5′–3′)	Restriction site/amino acid exchange
	Δ*aglH*	
1842	CCCACTGGGCCCGAGAGTGTTAGAAAGAGAAGA	*Apa*I
1843	GGTTATCGTAACCGTCAGGGATACGAGCATTATTTTCTCCTTCTT	
1844	ATGCTCGTATCCCTGACGGTTACGATAACCATCTAATGATATACA	
1845	CGCCGAGGATCCTCAACGTGGAATTCCCTCTCC	*Bam*HI
	*aglH*_FLAG_	
1755	GCGGGAATTCATGCTCGTATCCCTGCTTGGTATTC	*Eco*RI
1756	GGCGCTCGAGTTACTTGTCATCGTCCTTGTAGTCAATGATGGTTATCGTAACCGT TTG	*Xho*I-FLAG
	*aglH*_FLAG_ point mutation	
2740	GGTTTTACAGGGAAGGCTATAAATAAATTGACTAAG	D039A
2741	CATCATCCTTAGTCAATTTATTTATAGCCTTCCC	D039A
2742	CTTGGTCTACTTGATGCTATTTTCAACATAAGCCAG	D100A
2743	GTTGAAAATAGCATCAAGTAGACCAAGAAAACC	D100A
2758	GGGAAGGACATAAATGCATTGACTAAGGATGATGTTCC	K042A
2759	CCTTAGTCAATGCATTTATGTCCTTCCCTGTAAAACC	K042A
2760	CTTGATGACATTTTCGCCATAAGCCAGGCTACTAG	N103A
2761	CCTGGCTTATGGCGAAAATGTCATCAAGTAGACC	N103A
2764	CTATCCGGCTGCAACTTTTCCAGGAAATATAGGTAC	K218A
2765	TATTTCCTGGAAAAGTTGCAGCCGGATAGAAATTG	K218A
2766	CAATTTCTATCCGGCTAAAACTGCTCCAGGAAATATAGG	F220A
2767	AATAAAATAAGTACCTATATTTCCTGGAGCAGTTTTAGCCGG	F264A
2768	GATCGTCAATTTTTCCGAACGAGACTCCCTTAGCCCTAGTTTTAG	F264A
	*aglH*_FLAG_Yeast	
4125	AATCACTGCAGATGCTCGTATCCCTGCTTGG	*Pst*I
4126	GGCGGAATTCTTACTTGTCATCGTCGTCCTTGTAGTCAATGATGGTTATCGTAACCGTTTG	*Eco*RI-FLAG

Mutated nucleotides and restriction sites are *underlined*

### Transformation and selection of *S. acidocaldarius* mutants

Competent cells were obtained according to the protocol of Kurosawa and Grogan (Kurosawa and Grogan 2005). Aliquots containing 400–600 ng of methylated plasmids (pSVA1229 or pSVA1246) were mixed with 50 µl of competent MW001 cells and incubated for 5 min on ice. Electroporation was performed using a Gene pulser II (Bio-Rad, USA) with the input parameters 1.25 kV, 25 mF, 1000 W in 1 mm cuvettes. Immediately after the pulse, 50 µl of a 2 × concentrated recovery solution (1% sucrose, 20 mM β-alanine, 20 mM malate buffer pH 4.5, 10 mM MgSO_4_) was added and the samples were incubated for 30 min at 75 °C under mild shaking conditions (150 rpm). Prior to plating, additionally 100 µl of heated 2x-concentrated recovery solution was added, and 2 × 100 µl was spread on gelrite plates containing Brock medium supplemented with 0.1% NZ-amine and 0.1% dextrin, lacking uracil. After incubation for 5–7 days at 75 °C, large brownish colonies were used to inoculate 50 ml of first selection Brock medium containing 0.1% NZ-amine. After 3 days of incubation at 75 °C, each culture was screened for the presence of the integrated plasmid by PCR. Positively tested isolates were inoculated in Brock medium (0.1% NZ-amine and 0.1% dextrin) and grown until an OD_600_ of 0.4 was reached. 40 µl of aliquots were then spread on second selection plates, where the recombination step was selected by the presence of 5-fluoroorotic acid (5-FOA) and uracil. After incubation for 5–7 days at 75 °C, differently sized colonies were picked and streaked onto new second selection plates to ensure single colony formation. Colonies were screened by PCR for the presence or the deletion of *saci0093* (*aglH)*, using the outer primers (1842 and 1845).

### Transformation and selection of the deletion mutant in YPH 499 *alg*7::HIS3/GAL1-*alg*7

The YPH 499 strain (Mat a; *ura*3–52; *lys*2–801^amber^; *ade*2–101^ochre^; *trp*1-∆63; *his*3-∆200; *leu*2-∆1) (Sikorski and Hieter 1989) has been used to replace the native *alg7* promoter with a selection marker/promoter HIS3/GAL1 cassette, resulting in strain YPH 499 *alg*7::HIS3/GAL1-*alg*7 (Mazhari-Tabrizi et al. 1999). The introduction of the HIS3/GAL1 cassette eliminated the strain’s auxotrophy for histidine and placed the *alg7* gene under the regulation of GAL1. YPH499 was transformed with the plasmid pSVA1212, as described previously (Eckert et al. 1998) and inoculated on SD medium plates, lacking uracil and histidine.

### Immunostaining of the AglH_FLAG_ point mutations expressed in *S. cerevisiae*

Cell pellets of the same OD_600_ from 5 ml of SGR medium cultures of YPH499-HIS-GAL-ALG7 transformed with the AglH complementation plasmids (Table 3) were resuspended in 1 ml of buffer A (100 mM NaCl, 100 mM Tris–HCl, 1 mM EDTA, pH 8) and lysed by 20 min sonification with an intensity of 60% and an interval 20 s (Bandelin Sonopuls). Unbroken cells were removed by centrifugation at 3000×*g* at 4 °C for 20 min. The supernatant was centrifuged in a Beckman Coulter Optima Max-XP Ultracentrifuge at 120,000×*g* at 4 °C for 45 min to obtain the membrane pellet. The membrane pellet was resuspended in 0.5 ml of buffer A. Aliquots of 30 µl were loaded on an 11% SDS-PAGE and run at 100 V. The expression of AglH_FLAG_ was analysed by Western immune blotting using the primary antibody anti-DYKDDDDK (Carl Roth, Germany) and an anti-rabbit IgG–alkaline phosphatase-coupled antibody (Sigma Aldrich, St Louis, USA). Chemifluorescence was measured in a Fujifilm LAS-4000 Luminescent image analyzer (Fujifilm, Duesseldorf, Germany).

**Table 3:** Plasmids used in this study

Plasmid	Genotype	References
pUC18	Cloning vector; Amp^r^	Yanisch-Perron et al. (1985)
pSAV407	Gene targeting plasmid, pGEM-T Easy backbone, *pyr*EF cassette of *S. solfataricus*	Wagner et al. (2012)
pSVA1229	Δ*aglH* cloned into pSVA407	This study
pSVA1246	*aglH*_STREP_ cloned into pSVA407	This study
pRS426 MET	Gene targeting and expression vector	Mumberg et al. (1995)
pSVA1212	*aglH*_FLAG_ (Saci0093) cloned into pRS 426 MET with *Xho*I, *Eco*RI	This study
pSVA1247	Based on pSVA1212 containing *aglH*-_**D039A-**_FLAG	This study
pSVA1248	Based on pSVA1212 containing *aglH*-_**D100A**_-FLAG	This study
pSVA1258	Based on pSVA1212 containing *aglH*-_N103A_-FLAG	This study
pSVA1259	Based on pSVA1212 containing *aglH*-_K218A_-FLAG	This study
pSVA1260	Based on pSVA1212 containing *aglH*_Δ218-329_	This study
pSVA1261	Based on pSVA1212 containing *aglH*-_K042A_-FLAG	This study
pSVA1262	Based on pSVA1212 containing *aglH*-_F264A_-FLAG	This study
pSVA1263	Based on pSVA1212 containing *aglH*-_F220A_-FLAG	This study
pUC18_*aglH*_FLAG_	Base on pUC18 containing *aglH* from *S. acidocaldarius*	This study

## Results

### Identification of AglH, a predicted UDP-GlcNAc-1-phosphate:dolichyl phosphate GlcNAc-1-phosphotransferase

**Fig. 1:**
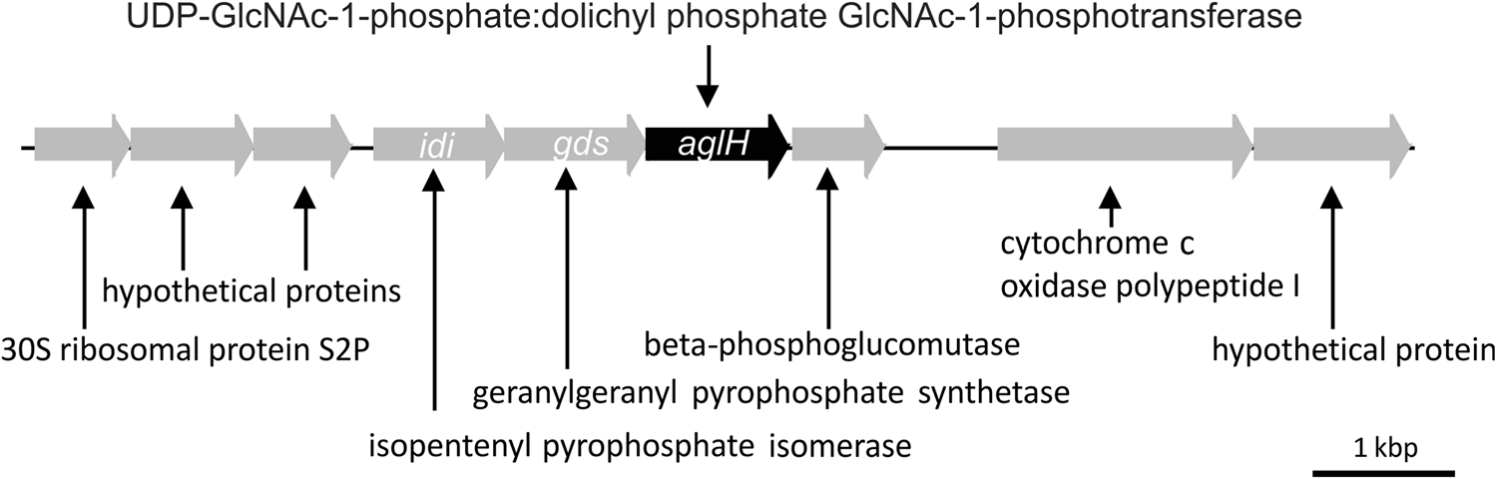
Physical map of the gene region adjacent to *algH* of *S. acidocaldarius*. Illustrated are the genes *Saci0088* until *Saci0096*. The gene *aglH* displayed in* black* (*saci0093, SACI_RS00435*) encodes the UDP-GlcNAc-1-phosphate:dolichyl phosphate GlcNAc-1-phosphotransferase. The genes *idi* (*saci0091*) and *gds* (*saci0092*) are involved in the isoprenoid lipids biosynthesis

To identify the enzyme required for the first step of the protein *N*-glycosylation pathway in the thermoacidophilic crenarchaeon *S. acidocaldarius,* its genome was analysed for the presence of eukaryotic, bacterial, and archaeal homologues of the UDP-GlcNAc-1-phosphate:dolichyl phosphate GlcNAc-1-phosphotransferase Alg7/Dpagt1, WecA, and AglH, respectively. This search identified *saci0093* (*SACI_RS00435*) encoding a predicted UDP-GlcNAc-1-phosphate:dolichyl phosphate GlcNAc-1-phosphotransferase, which shares 27% amino acid sequence identity with the yeast Alg7 and the human Dpagt1, 31% with AglH from the euryarchaeon *M. voltae* and 26% with WecA of *E.coli*. *Saci0093* is located in a gene locus downstream of genes whose products are predicted to be involved in the early steps of isoprenoid lipid biosynthesis (Fig. 1). The gene s*aci0091* (*SACI_RS00425*, *idi*) codes for an isopentenyl diphosphate delta-isomerase, which is necessary for the biosynthesis of isoprenoid compounds via the mevalonate pathway (Boucher et al. 2004). The corresponding protein from *S. shibatae* is an isopentenyl-diphosphate delta-isomerase that catalyses the interconversion between two active units for isoprenoid biosynthesis, isopentenyl diphosphate (IPP) and dimethylallyl diphosphate (DMAPP) (Nakatani et al. 2012; Yamashita et al. 2004). *Saci0092* (*SACI_RS00430*, *gds*) encodes a bifunctional geranylgeranyl pyrophosphate synthase that synthesizes both farnesyl pyrophosphate (FPP) and geranylgeranyl pyrophosphate (GGPP) (Ohnuma et al. 1998; Ohnuma et al. 1996). GGPP is an intermediate for the biosynthesis of many isoprenoid compounds like carotenoids, geranylgeranylated proteins, as well as archaeal ether-linked membrane lipids. Furthermore, GGPP acts as a precursor in the proposed DolP biosynthesis pathway (Guan et al. 2011). Analyses of the *Sulfolobales* transcriptome revealed a polycistronic mRNA of s*aci0092* and s*aci0093* (Wurtzel et al. 2010), indicating a coordinated gene regulation and/or expression for the biosynthesis of DolP and the DolPP-GlcNAc primer in the *N*-glycosylation process.

### AglH from *S. acidocaldarius* shows high similarity in primary sequence and transmembrane topology with eukaryal and bacterial UDP-GlcNAc-1-phosphate:polyprenyl phosphate GlcNAc-1-phosphotransferases

**Fig. 2:**
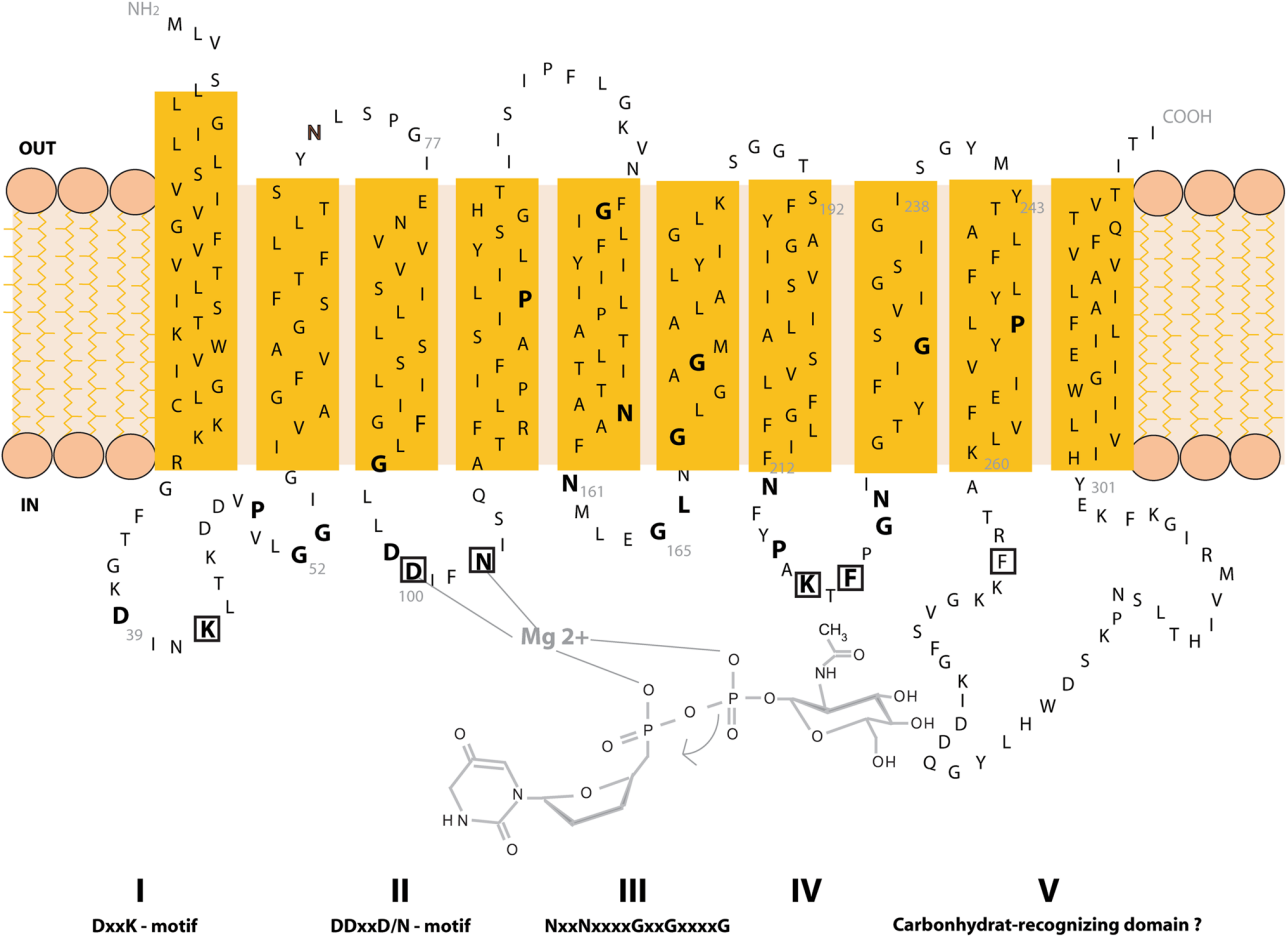
Topology model of AglH from *S. acidocaldarius*. The topological model was derived using the TMHMM server (http://www.cbs.dtu.dk/services/TMHMM/) and PSIPRED protein structure prediction server (http://bioinf.cs.ucl.ac.uk/psipred/). Conserved amino acids (see alignment Fig. 3) are shown in *bold*. *Numbers* indicate internal cytoplasmic loops I to V, as well as their conserved motifs. Proposed catalytic interaction with UDP-GlcNAc is displayed in *light grey*. *Boxed* amino acids were replaced by alanine

**Fig. 3:**
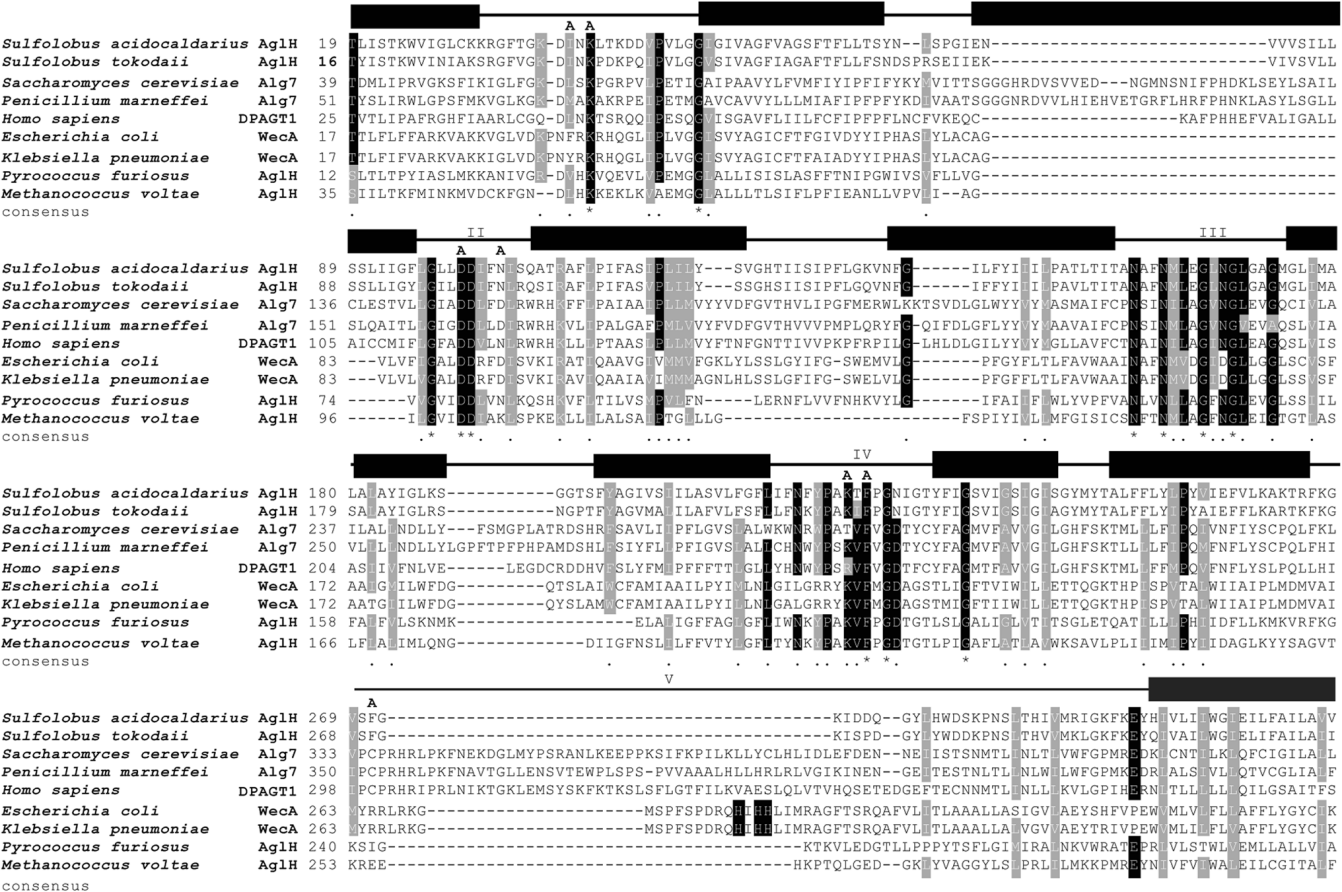
Alignment of archaeal, bacterial, and eukaryal UDP-GlcNAc-1-P transferase orthologs. Orthologs were identified using BLAST with *S. acidocaldarius* Saci0093 (AglH). Protein sequences were aligned with the ClustalW program. The partial alignment is shown for the region encompassing the conserved motifs DxxK (CL I), DDxxN/D (CL II) and NxxNxxxxGxxGxxxxG (CL III). Conserved amino acids are indicated with asterisks. The boundaries of transmembrane domains (*filled boxes*) and cytosolic loops (*lines*) are shown on *top* of the alignment. The replacement of selected amino acids from AglH of *S. acidocaldarius* by alanine (**a**) are indicated above the sequence

Topology prediction of AglH (Saci0093) in combination with the known primary sequence revealed high levels of similarities with eukaryal as well as bacterial GlcNAc-1-phosphotransferases. AglH possesses ten transmembrane (TM) helices separated by five internal and four external hydrophilic loops (Fig. 2). A similar topology was shown for the eukaryal Alg7 enzyme with ten predicted TM domains, while bacterial WecA typically exhibits ten to eleven TM domains (Anderson et al. 2000; Lehrer et al. 2007). For the archaeal homologue from *M. voltae* only seven TM domains have been predicted (Shams-Eldin et al. 2008). An alignment of AglH with the eukaryal, bacterial, and archaeal orthologues illustrated high amino acid similarity within each of the five cytoplasmic loops, indicating a possibly conserved function across the three domains of life (Fig. 3).

In the cytoplasmic loop (CL) I, a conserved D_39_xxK/N motif is detected (Figs. 2, 3). In the CL II of Saci0093, a D_99_D_100_xxN_103_ motif is present, similar to the conserved motif of eukaryal and bacterial DDxxD motifs (Fig. 3). This conserved motif is potentially involved in Mg^2+^ cofactor binding (Amer and Valvano 2002; Lloyd et al. 2004; Xu et al. 2004). Within the fifth TMD, the CL III, and the six TMD, a long N_158_xxN_161_xxxxG_165_xxG_168_xxxxG_173_ motif is present. The two conserved aspartic residues have been mutated in the *E. coli* WecA_DD156/159GG_, which resulted in only 3% of the wt transferase activity (Amer and Valvano 2002). The impaired function in vivo and the reduction of enzyme activity in vitro suggest the importance of this motif for the catalytic activity. This motif, in combination with the DDxxK/N motif of the second cytoplasmic loop, resembles the characteristics of a Walker B motif (Amer and Valvano 2002). The large predicted CL V is thought to be involved in the recognition of the carbohydrate moiety of the UDP-GlcNAc donor. In the respective loop of bacterial WecA, a conserved histidine residue is located within a less conserved short-sequence motif HIHH (Amer and Valvano 2001; Anderson et al. 2000). Replacement of the highly conserved His_279_ residue with serine rendered WecA unable to restore O7 production in an *E. coli wec*A::Tn10 strain (Lehrer et al. 2007). Unlike bacteria, eukaryal and archaeal orthologues are missing the HIHH motif. If the functional interpretation for WecA is correct, this raises the question of how the carbohydrate moiety of the UDP-GlcNAc donor is being recognized in these two domains of life (Fig. 3).

Collectively, the sequence features for AglH are entirely consistent with it being a UDP-GlcNAc-1-phosphate:polyprenyl phosphate GlcNAc-1-phosphotransferase participating in the *N*-glycosylation pathway.

### AglH is essential for viability in *S. acidocaldarius*

Based on the obvious conservation of the different motifs within the cytoplasmic loops as well as the characteristic topology profile of AglH (Saci0093), we propose that *algH* indeed encodes a UPD-GlcNAc-1-P transferase initiating the protein *N*-glycosylation process by transferring GlcNAc-1-P from UDP-GlcNAc to DolP. To verify his hypothesis, we designed the plasmid pSVA1229, incorporating the up- and downstream region enclosing Δ*aglH*, to create a markerless deletion mutant of *aglH* in *S. acidocaldarius* by homologues recombination. Integration of this plasmid in *S. acidocaldarius* MW001 was confirmed by PCR using primers encompassing the flanking regions of the target gene. A second homologous recombination, enforced by the addition of 5-fluoroorotic acid, resulted in the segregation of the plasmid, generating either the wild-type stain or the preferred Δ*aglH* deletion strain. Screening of more than 150 different colonies by PCR using primers derived from the flanking regions revealed only the presence of wild-type genetic organization. This result suggests that AglH and *N*-glycosylation process is essential for the archaeon. Polar effects cannot be completely excluded; however, they are unlikely, given the fact that the integration of the plasmid could be achieved and this did not alter the archaeal phenotype. Furthermore, all essential genes for isoprenoid lipid biosynthesis are located upstream of the *aglH*. The segregation of the plasmid would not disturb the genetic neighbourhood, as the integrants did not show any evident alteration in growth.

**Fig. 4:**
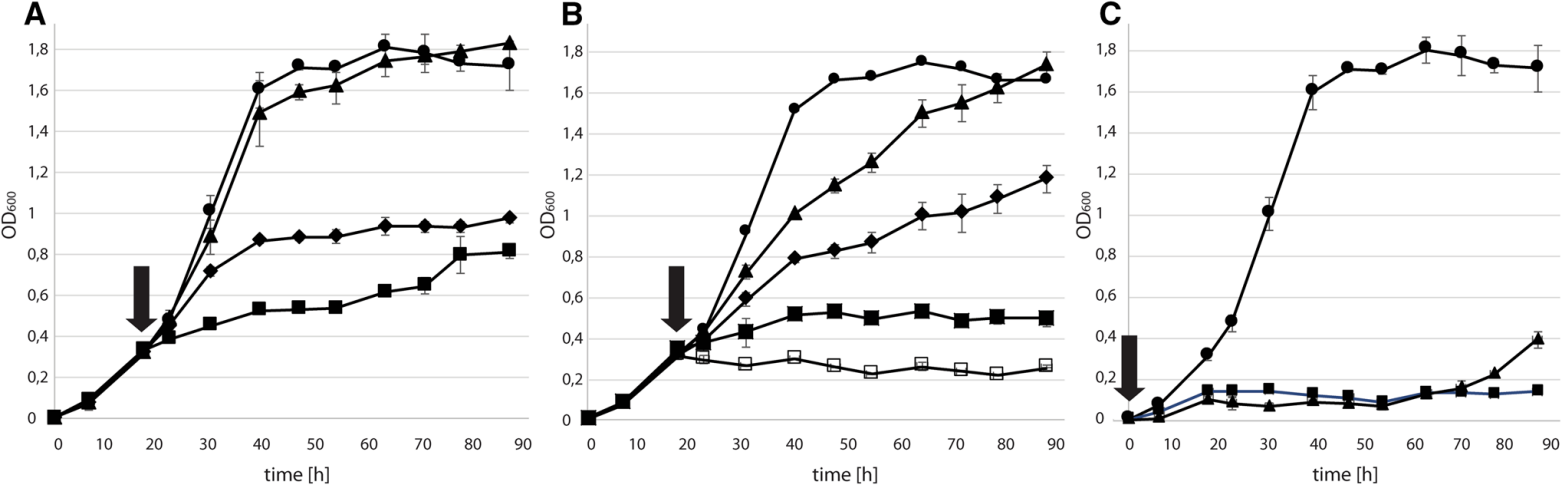
Effect on the cell growth of *S. acidocaldarius* by the *N*-glycosylation inhibiting antibiotics bacitracin and tunicamycin. **a** Different concentrations of bacitracin: 0 mM (*filled circle*), 0.6 mM (*filled triangle*), 1.2 mM (*filled diamond*), 2.4 mM (*filled square*) mM were added after reaching exponential phase, indicated by *black arrow*. **b** Different concentrations of tunicamycin: 0 (*filled circle*), 2 µg/ml (*filled triangle*), 6 µg/ml (*filled diamond*), 16 µg/ml (*filled square*), 32 µg/ml (*open square*) were added after reaching exponential phase. **c** Cell growth without antibiotics (*filled circle*), with 1.2 mM bacitracin (*filled square*), or 6 µg/ml tunicamycin (*filled triangle*), antibiotics were added directly after inoculation of the culture, indicated by *black arrow*

To underline the essentiality of AglH activity, we explored the growth of *S. acidocaldarius* in the presence of antibiotics interfering specifically with the initiation of the *N*-glycosylation. Tunicamycin, a nucleoside antibiotic, resembles UDP-HexNAc residue and specifically inhibits UDP-HexNAc:polyprenol-P HexNAc-1-P transferases by blocking the active site (Esko and Bertozzi 2009). Bacitracin interferes with the dephosphorylation of isoprenyl pyrophosphate hindering the recycling of new isoprenyl phosphate lipid carrier (Stone and Strominger 1971). The addition of both antibiotics, after reaching early exponential growth phase, showed a dose-dependent reduction of growth of *S. acidocaldarius* (Fig. 4a, b). The addition of 0.3 mM bacitracin (*t*_d_ = 10.7 ± 0.6 h) did not significantly reduce the growth compared to the wt doubling time (*t*_d_ = 9.2 ± 0.9 h), whereas the addition of 1.2 and 2.4 mM bacitracin decreased the growth two- and fourfold (*t*_d_ = 18.5 ± 2.5 h; *t*_d_ = 42.1 ± 4 h), respectively. In addition, the addition of tunicamycin reduced the growth rate of *S. acidocaldarius*. Here, 2 µg/ml (*t*_d_ = 13.6 h ± 1.6) and 6 µg/ml (*t*_d_ = 16.4 h ± 1.2) led to a significant reduction of growth. Furthermore, the addition of 16 µg/ml increased the doubling time to 40.1 h ± 1.3 for the next 20 h. No growth was detected after 20 h in the samples treated with 16 µg/ml tunicamycin and with the two highest bacitracin concentrations. Interestingly, cells appeared larger compared to wt strain. Thus, the increased OD within the first hours may be attributed to an enlarged cell size. At 32 µg/ml no growth could be detected (Fig. 4b). The effect of the antibiotics inhibiting *N*-glycosylation and thereby inhibiting cell growth was the most obvious when the antibiotics were directly added after inoculation (Fig. 4c). Here, first the OD_600_ slightly increased to 0.1–0.15, then no growth could be detected over 70 h. Based on the reduced stability of the antibiotics (i.e. tunicamycin) at the growth condition (pH 3, 75 °C), the cells recover growth after 70 h.

### AglH restores growth of a conditional lethal *alg*7 yeast mutant in a complementation assay

**Fig. 5:**
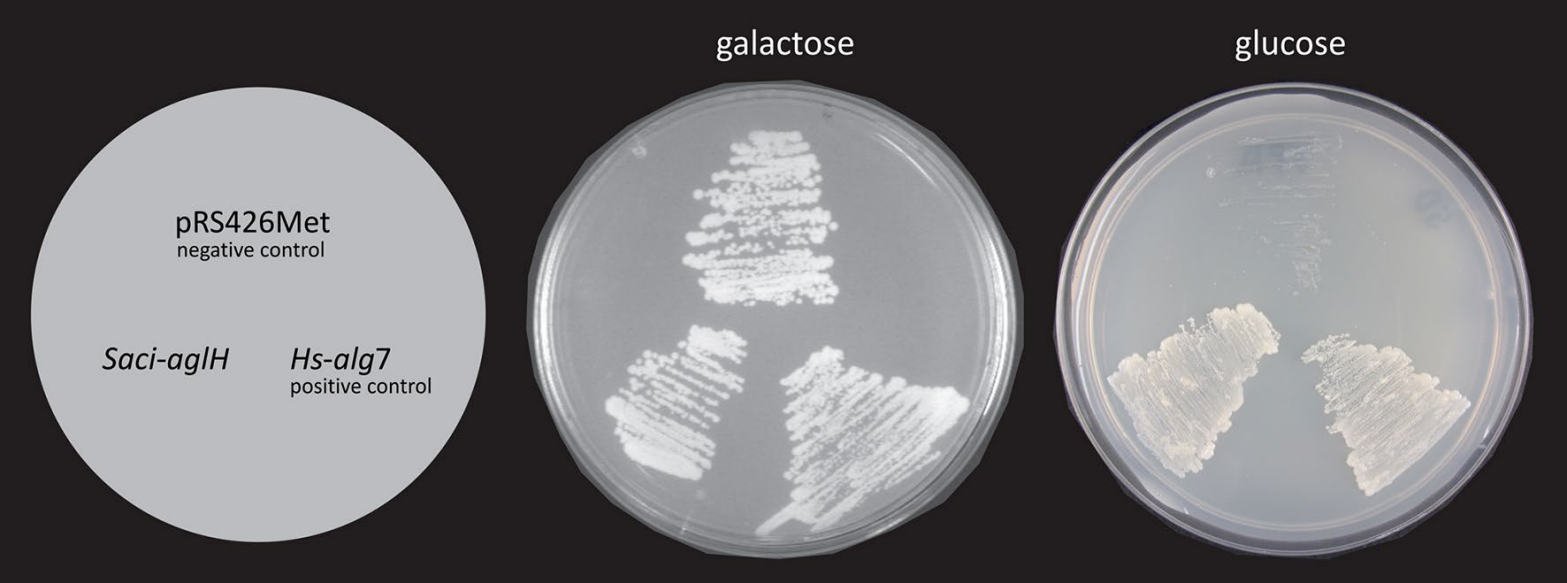
Rescue of a conditional lethal *alg*7 yeast mutant by the thermophilic *aglH* from *S. acidocaldarius.* YPH499-HIS-GALprom-ALG7 was transformed with the pRS426-METt plasmids carrying either the human ALG7 (*Hs*-*alg*7) or the archaeal Saci0093 (*Saci*-*aglH*). Transformed cells were streaked onto plates containing minimal medium lacking histidine and uracil and containing either galactose or glucose

To confirm the function of AglH, we tested whether this gene is able to complement a conditional lethal yeast *alg7* mutant, as has been successfully shown for the human *alg*7 (Eckert et al. 1998) and *aglH* of *M. voltae* (Shams-Eldin et al. 2008). The full length *algH* gene was cloned into a yeast shuttle vector pRS426Met (Mumberg et al. 1995), containing the selective marker *ura3* ^+^, resulting in the plasmid pSVA1212. After transformation of this plasmid into the conditional lethal yeast *alg*7 mutant, YPH499-HIS-GAL-ALG7 (Mazhari-Tabrizi et al. 1999), transformants were plated on SGR as well as SD medium lacking histidine and uracil. Using plates containing either SGR medium (containing galactose) or SD medium (lacking galactose), in which the endogenous yeast *alg*7 is expressed or repressed, respectively. Cells transformed with the empty pRS426Met vector served as a negative control and displayed growth only on galactose-containing plates, i.e. conditions under which endogenous *alg*7 was expressed (Fig. 5). Under *alg*7 repression conditions, this strain failed to grow. Strains containing the plasmid carrying the human *alg*7 (positive control) (Eckert et al. 1998) or the *aglH* gene displayed sustained growth on glucose-containing plates, demonstrating the ability of the gene product to functionally replace the essential function of the yeast Alg7 enzyme (Fig. 5). These results showed that *S. acidocaldarius* AglH is indeed functional in *S. cerevisiae* and able to suppress the lethal phenotype of *alg*7 by catalysing the transfer of GlcNAc-1-P onto DolP.

### Replacement of conserved amino acids resulted in a functional loss of AglH

**Fig. 6:**
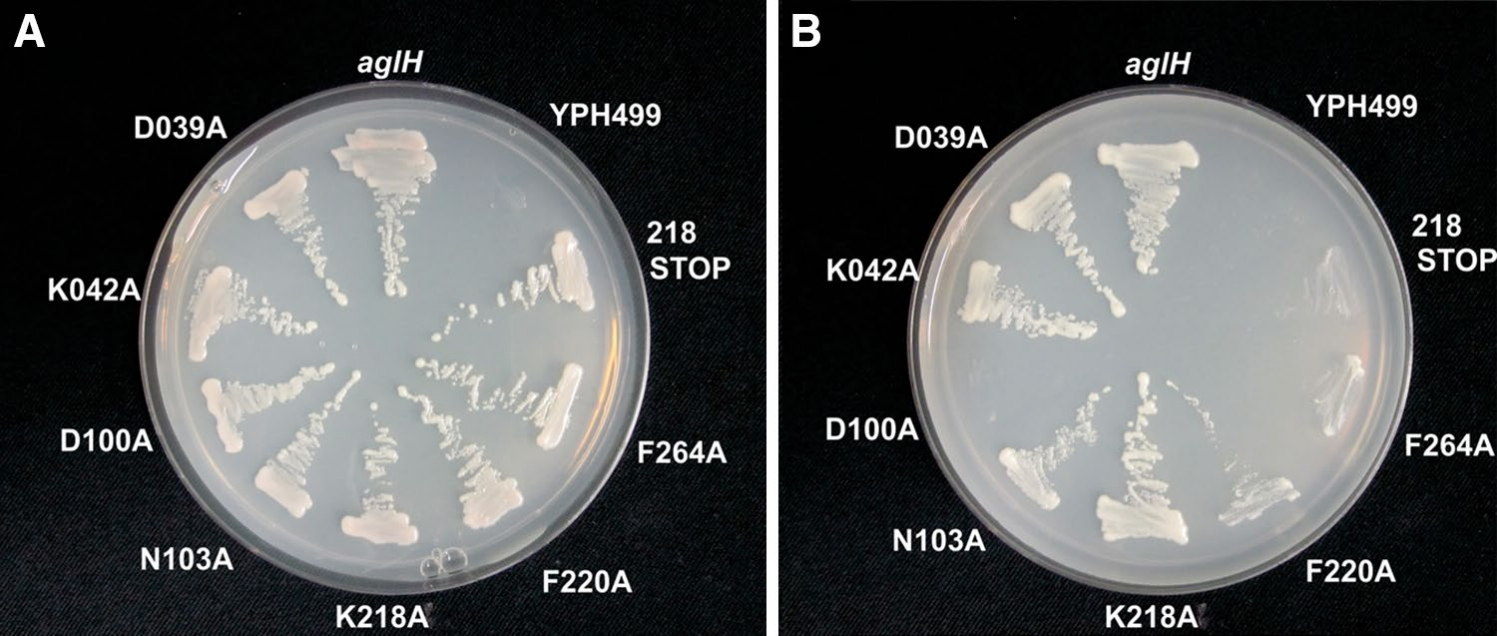
Functional characterization of AglH in YPH499-HIS-GAL-ALG7 by a complementation assay. The conditional lethal mutant YPH499-HIS-GAL-ALG7 was transformed with the pRS426-METt plasmid carrying either the archaeal *aglH* (Saci0093) or *aglH* with selected point mutations, resulting in the exchange of conserved amino acids to alanine. The transformed cells were then streaked onto plates containing minimal medium lacking histidine as well as uracil and containing either galactose (**a**) or glucose (**b**). The exchange of the conserved amino acids D_100_A, F_264_A resulted in a lethal, whereas the exchange of F_220_A resulted in a slow-growing phenotype of YPH499-HIS-GAL, under repression condition of the *alg*7 gene (**b**)

The complementation assay was employed to verify the functional importance of distinct conserved amino acid residues in the predicted cytoplasmic loops (CL) of *Saci*AglH. The residues mutated were D_39_ and K_42_ in CL I, D_100_ and N_103_ in CL II, K_218_ and F_220_in CL IV and F_264_ in CL V; all were replaced individually by alanine. Mutations were introduced in the plasmid containing the *aglH* (Saci0093) gene fused to sequences encoding a FLAG epitope tag. The AglH-FLAG fusion protein is fully functional and allows growth of the conditional lethal yeast mutants on SD medium (Fig. 6). Mutations in CL I (D_039_A and K_042_A) had no effect on the function of the AglHFLAG protein. Also N_103_A and K_218_A mutations in CL II and CL III did not affect growth of the yeast mutant. In contrast, the strain complemented with the D_100_A showed no growth under conditions that repressed the yeast *alg*7 gene, implying that D_100_ could be essential for catalysis. The F_220_A and F_264_A mutations in CL IV and CL V, respectively, had different effects. While the F_220_A mutant was substantially impaired in activity as indicated by very poor growth in the complementation assay (Fig. 6), the F_264_A mutant appeared inactive. To investigate the function of the C-terminal part of the enzyme, a stop codon was introduced at amino acid position 218 AglH_Δ218-329_.The insertion of a stop codon prevented the ability to functionally complement the Alg7 phenotype. These results showed that the amino acids D_100_, F_220_ and F_264_ in AglH are important for successful complementation of the lethal phenotype of YPH499-HIS-GAL-ALG7.

### Exchange of conserved amino acid residues did not change the expression level of AglH_FLAG_

**Fig. 7:**
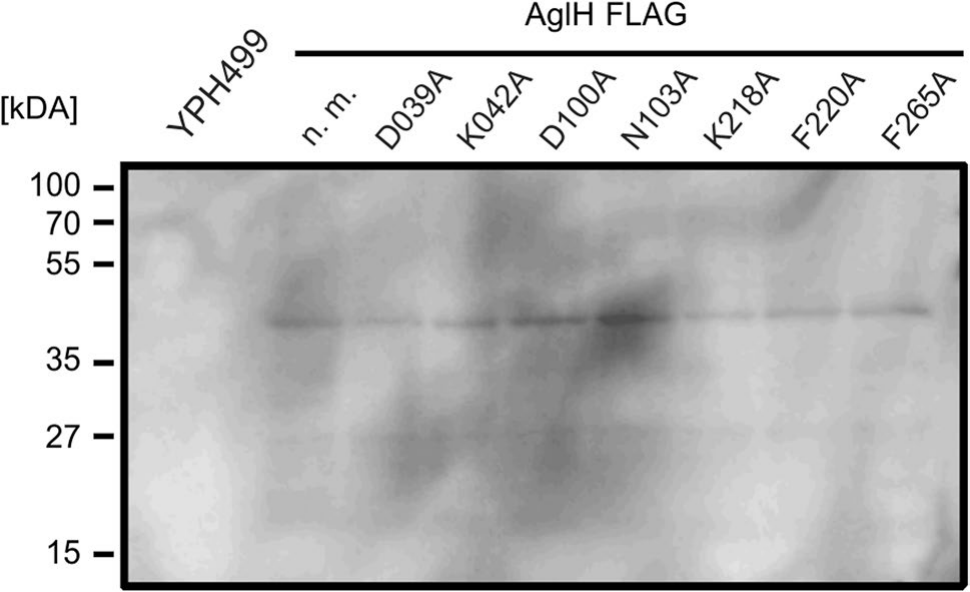
Detection of the protein expression from the derived AglH_FLAG_ point mutation by Western immunoblotting. Equivalent amounts of cells from YPH499 background strain (*lane 1*) or the complemented strains with the AglH_FLAG_ expression vector (*lane 2–9*) were separated by 11% SDS-PAGE and immunoblotted with antibodies raised against the FLAG-tag epitope. The non-mutated AglH_FLAG_ (n. m.) showed a similar expression level as the different AglH_FLAG_ point mutations (*lane 3–9*)

To ensure that the AglH_FLAG_ derivatives were expressed and to eliminate the possibility of reduced protein stability due to the amino acid replacements, protein expression was investigated. The membrane fraction of each of the mutated AglH_FLAG_ transformed strains was examined by immuno blotting (Fig. 7). AglH was detected in the wild-type and all mutated AglH_FLAG_ versions, showing that the lack of function observed in Fig. 7 for the D_100_A, F_220_A, and F_264_A mutations is not caused by reduced protein expression or degraded protein. The observed molecular weight of AglH_FLAG_ corresponds to the calculated molecular weight of 36.6 kDa.

## Discussion

Over the last decade, research on archaeal *N*-glycosylation pathways has revealed the diversity of the glycosylation process in terms of the involvement of specific GTases, as well as the structural and compositional complexity of archaeal *N*-glycans (Jarrell et al. 2014). In addition, these studies demonstrated a significant role of glycosylation for the stability of S-layer proteins as well as for the stability and the assembly of the archaellum, the archaeal motility structure (Calo et al. 2010; Yurist-Doutsch et al. 2008). In the thermoacidophilic crenarchaeon family *Sulfolobales*, most if not all surface-exposed proteins (Palmieri et al. 2013), including the S-layer protein (Meyer et al. 2011), the archaellin (Meyer et al. 2013; Meyer et al. 2011), and sugar binding proteins (Elferink et al. 2001), are post-translationally modified by *N*-glycosylation. Defects in *N*-glycan biosynthesis resulted in a significant effect of the growth under elevated salt concentrations as well as a reduced motility (Meyer et al. 2011, 2013).

To understand the *N*-glycosylation in *S. acidocaldarius* in more detail, we searched for the enzyme initiating this process. We identified *aglH* (*saci0093*), coding for a UDP-GlcNAc-1-phosphate:dolichyl phosphate GlcNAc-1-phosphotransferase, which shows high similarities in the overall topology as well as in the amino acid sequence with the eukaryotic Alg7, bacterial WecA, and archaeal homologs (Fig. 3). Based on these structural and sequence similarities, we analysed whether AglH acts also as a functional homolog by complementation of a conditional *alg7* yeast deletion mutant. Such complementation has been successfully demonstrated previously with the euryarchaeal AglH (Mv1751) from *M. voltae* (Shams-Eldin et al. 2008). Here, we showed that also the thermophilic AglH enzyme complements defects in the biosynthesis of pyrophosphate-linked GlcNAc-isoprenoid units of Eukarya. Although the AglH from *S. acidocladarius* is adapted to high temperatures of around 75 °C, this enzyme is still able to complement the conditional lethal *alg*7 yeast mutant restoring the *N*-glycosylation process at 28 °C.

In addition to the ability of AglH to function in Eukarya, we could demonstrate that the substitution of conserved amino acid residues D_100_, F_220_, and F_264_ leads to loss of function in the in vivo complementation assay in yeast (Fig. 6). The inability of the AglH D_100_A mutant to complementing the conditional yeast mutant is consistent with the substitution of the two aspartic acids DD_90/91_GG in WecA of *E. coli,* where the mutant was impaired in its activity in vitro and in an in vivo complementation system (Amer and Valvano 2002). It is proposed that the DDxxN/D motif and the conserved motif (NxxNxxxGxxGLxxG) of CL III catalyse the formation of the diphosphate linkage of DolPP-GlcNAc (Fig. 3), as the two conserved motifs show sequence similarity to the Walker B motif (Amer and Valvano 2002). The substitution of the highly conserved F_220_ located in the CL IV leads to a drastic reduction in the ability of the AglH to functionally replace Alg7 in yeast. Although little growth could be detected on glucose-containing plates (non-permissive), which is most likely in response to a small amount of Alg7 remaining from the pre-culture on SRG medium, a significantly reduced activity of the AglH F_220_A is obvious. The data for this mutant align well with the corresponding F_249_L in eukaryotic UDP-GlcNAc:dolichol-P GlcNAc-1-P transferase (GTP), which resulted in a loss of more than half of the enzymatic activity (Dal Nogare et al. 1998). The function of the less conserved F_264_, is unclear, but the whole CL V might be involved in the in recognizing the nucleotide-activated sugar donor. Apart from proposed function of the CL V, the overall basic loop might be involved in the oligomerization or interaction with cytosolic GTases. Recently, Alg13/Alg14, which catalyses the second step in eukaryotic *N*-glycan biosynthesis, was found to interact with Alg7. This interaction tethers the soluble Alg13/Alg14 GTases to the membrane of the ER (Lu et al. 2011), which allows the enhanced biosynthesis of the *N*-glycan by clustering the GTase reactions. As *S. acidocaldarius* possesses also a chitobiose core as the *N*-linking unit at the reducing terminus of its *N*-glycan like Eukarya (Peyfoon et al. 2010; Zahringer et al. 2000), a comparable assembly process might also be present in this archaeon. However, bioinformatics searches for an Alg13/Alg14 ortholog in *S. acidocaldarius* failed to identify an *N*-acetylglucosamine transferase.

Deletion of *aglH* was not possible, implicating an essential role of this gene for the viability of *S. acidocaldarius*. This result is consistent with the inability to delete the key enzyme AglB of the *N*-glycosylation process, catalysing the transfer of the oligosaccharide onto target proteins (Meyer and Albers 2014). Treatment with antibiotics directly interfering with the function of AglH function furthermore strengthened the importance of AglH for cell survival (Fig. 4). Studies of the membrane-bound pyrophosphatase SepP (Saci1025) in *S. acidocaldarius,* recycling DolPP back into DolP precursor, revealed the necessity of this DolP cycle (Meyer and Schafer 1992). Here, supplementation of bacitracin interfering with the function of SepP into the growth medium led to the death of the cells, consistent with our findings (Fig. 4). Also treatment with tunicamycin has been previously shown to result in a reduced growth, a gradual increase in cell size, and cell death in *S. acidocaldarius* (Hjort and Bernander 2001). Furthermore, treatment of tunicamycin resulted also in a reduction in the relative amount of glycosylated proteins (Grogan 1996), underlining that this process is indeed important for generating *N*-glycans. However, at that time, no analyses on the lipid-linked glycans confirming the presence of DolPP-linked glycans in *S. acidocaldarius* or other crenarchaeota have been performed. Analyses of the lipid-linked glycans in euryarchaea revealed the presents of DolP rather than DolPP-linked oligosaccharide (Chaban et al. 2009; Guan et al. 2010; Taguchi et al. 2016). In *Hfx. volcanii* and in *M. voltae*, the enzymes AglK and AglJ have been shown to initiate the *N*-glycosylation process by creating DolP-linked glycans, respectively (Kaminski et al. 2010; Chaban et al. 2009; Larkin et al. 2013). Interestingly, besides AglK, a homolog of AglH/Alg7/Dpagt1 has been identified in *M. voltae* (Chaban et al. 2006). A complementation assay in yeast, as it was done in this study, revealed that this AglH is able to replace the function of the eukaryal Alg7, which transfers GlcNAc-1-P from UDP-GlcNAc to DolP, yielding DolPP-GlcNAc (Shams-Eldin et al. 2008). Therefore, it was previously proposed that AglH initiates the *N*-glycosylation in *M. voltae*. However, lipid analyses as well as a detailed biochemical characterisation of AglK and AglH revealed that DolPP-GlcNAc in *M. voltae* is not used for the *N*-glycan assembly, as only DolP-linked oligosaccharides have been detected (Larkin et al. 2013). The study furthermore showed that AglK and AglC, the second enzyme of the *N*-glycosylation pathway, synthesize DolP-GlcNAc-Glc-2,3-diNAcA. This DolP-linked disaccharide is transferred by AglB onto a target peptide in vitro, proving that DolP not only can act as an acceptor but also is used as the glycan donor in *M. voltae* (Larkin et al. 2013).

Here, we proposed that in contrast to euryarchaea the *N*-glycan in crenarchaeon *Sulfolobus* is assembled on DolPP, similar to Eukarya. In fact, two recent studies confirmed the presence of the DolPP-linked *N*-glycans in *Sulfolobales* (Guan et al. 2016; Taguchi et al. 2016). Interestingly the study of Taguchi et al. underlined the difference of the lipid carrier between cren- and euryarchaea, as in all analysed euryarchaea DolP-linked oligosaccharides were present, whereas in the crenarchaea *P. calidifontis* and *S. solfataricus* lipid-linked oligosaccharides were assembled on DolPP backbone (Taguchi et al. 2016). The use of DolPP like in Eukarya emphasises the close evolutionary relationship between Crenachaeota and Eukarya and strengthens the proposed origin of Eukarya from within the TACK super phylum, comprising also the crenarchaeota (Archibald 2008; Guy and Ettema 2011). Interestingly, a newly characterised archaeal phylum, termed Lokiarchaeota, proposed to be one of the deepest branched archaea forms a monophyletic group with eukaryotes in phylogenomic analyses. Within the genomes of these Lokiarchaeota subunits of the eukaryal oligosaccharyltransferase complex are found, i.e. OST3/OST6 (Spang et al. 2015), which had not been found in Archaea so far. This indicates that the archaeon, from which the eukaryotic cell emerged, might have possessed an already highly developed protein *N*-glycosylation pathway.

With this study, we have identified the enzyme that catalyses the first step of the protein *N*-glycosylation process in *S. acidocaldarius*. This will facilitate a more detailed understanding of the archaeal version of this posttranslational modification. Future analyses will be directed to identify additional components of the thermophilic crenarchaeal protein *N*-glycosylation process.
